# Oxidative Stress Induces Mouse Follicular Granulosa Cells Apoptosis via JNK/FoxO1 Pathway

**DOI:** 10.1371/journal.pone.0167869

**Published:** 2016-12-09

**Authors:** Qiannan Weng, Zequn Liu, Bojiang Li, Kaiqing Liu, Wangjun Wu, Honglin Liu

**Affiliations:** 1 College of Animal Science and Technology, Nanjing Agricultural University, Nanjing, China; 2 Liaoning Province of Animal Product Safety Supervision, Shenyang, China; Institute of Zoology Chinese Academy of Sciences, CHINA

## Abstract

The c-Jun N-terminal protein kinase (JNK) plays an important role in the regulation of cell apoptosis. Forkhead box O (FoxO) transcription factors are involved in diverse biological processes, including cellular metabolism, cell apoptosis, and cell cycle. However, the JNK/FoxO1 pathway involved in the process of apoptosis induced by oxidative stress remains to be elucidated. Here, we demonstrated that the JNK activity significantly increased in response to oxidative stress in mouse follicular granulosa cells (MGCs). SP600125, a selective JNK inhibitor, attenuated the oxidative stress-induced MGCs apoptosis. Oxidative stress enhanced the FoxO1 nuclear translocation by activating the JNK activity. Moreover, JNK mediated the dissociation of FoxO1 from 14-3-3 proteins in MGCs after the treatment with H_2_O_2_. Finally, oxidative stress up-regulated the expression of FoxO1 via JNK mediation of FoxO1 self-regulation in MGCs. Taken together, our findings suggest that JNK/FoxO1 is involved in the regulation of oxidative stress-induced cell apoptosis in MGCs.

## Introduction

Folliculogenesis is a complex process which consists of primordial, primary, secondary, antral follicles and ovulation. Most of the follicles undergo degenerative process during ovarian follicle development in mammalian, which is called follicular atresia [1]. Follicular compartments comprise oocyte, theca and granulosa cells. Research indicated that granulosa cells apoptosis can cause the follicular atresia [2]. Moreover, several studies showed that follicular granulosa cells have a crucial role in oocyte potency in sheep [3], mouse [4], porcine [5], goat [6]and rat [7]. Therefore, follicular granulosa cells play an important function during female reproduction.

c-Jun N-terminal protein kinases (JNKs) belong to the mitogen-activated protein kinase (MAPK) family, which play a critical role in the regulation of stress, cell differentiation and cell apoptosis [8]. To date, 10 different splice variants of JNKs encoded by three genes, namely JNK1, JNK2, and JNK3, have been cloned and identified [9]. JNK1 and JNK2 are broadly expressed in all tissues, whereas JNK3 is mainly expressed in neuronal, testicular, and heart tissues [10, 11]. JNKs activation is mediated by MKK4 and MKK7 through phosphorylation of Thr183 and Tyr185 [12–14]. After JNKs are activated, they phosphorylate the serine residues 63 and 73 at c-Jun N-terminal, activating c-Jun and enhancing its transcriptional activity [14]. The activated JNKs also phosphorylate and regulate the activity of other transcription factors including ATF2, Elk-1, Trp53 and c-Myc [15, 16]. JNKs play an important role in death receptor-mediated such as those of TNF-a, TRAIL, and FasL, as well as mitochondria-mediated apoptotic events [9].

The forkhead box O (FoxO) transcription factor family plays an important role in the conserved pathway downstream, including the serine/threonine protein kinase B (PKB)/Akt, insulin, and insulin-like growth factor receptors [17, 18]. The FoxO family in mammals contains four members (FoxO1, FoxO3, FoxO4, and FoxO6) that share high protein homology [19]. A previous study showed that FoxO1 and FoxO4 are highly expressed in adipose and skeletal muscle tissues, whereas FoxO3 is abundant in the brain, heart, kidney, and spleen [20]. In addition, FoxO6 plays a crucial role in the nervous system, being mainly expressed in the development of adult brain [21]. FoxOs participate in various cellular processes, including cell proliferation, cycle, and apoptosis, whose functions are regulated by Akt and other signal pathways [22]. FoxO1 regulates *Bim* gene expression by binding to its promoter in oxidative stress-induced apoptosis [23]. Moreover, FoxO1 induces cell apoptosis via modulating Puma expression in oxidative stress-induced MGCs [24].

A previous study showed that 14-3-3 proteins are involved in cell cycle control, proliferation, apoptosis, disease and survival pathways [25]. The 14-3-3 proteins can interact with target proteins and alter their activity and intracellular localization [26]. Furthermore, the 14-3-3 proteins regulate pro- and anti-apoptotic through Bcl-2 and P53 pathways [26]. For example, in the presence of a survival factor, 14-3-3 proteins bind to BAD, ultimately facilitating its inactivation [27]. Moreover, it has been described that the phosphorylation of 14-3-3 proteins dissociates Bax, resulting in translocation of Bax to the mitochondria and apoptosis [28].

In the present study, in order to demonstrate that JNK is involved in the MGCs apoptosis induced by oxidative stress, we first investigated the JNK activity and the effects of SP600125 on cell apoptosis in oxidative stress-induced MGCs. In addition, we analyzed the expression of cytoplasmic and nuclear FoxO1 and its nuclear location in MGCs after JNK inhibition with SP600125. Finally, we demonstrated that SP600125 affected the interaction between FoxO1 and 14-3-3 proteins, as well as FoxO1 self-regulation in hydrogen peroxide (H_2_O_2_)-treated MGCs.

## Materials and Methods

### Ethic statement

All the animals were protected according to the guidelines of the Nanjing Agricultural University Animal Care and Ethics Committee. Mice were housed in a temperature-controlled room with proper darkness-light (12D:12L) cycles with lights on from 07:00 to 19:00, fed with a regular diet, and maintained under the care of the Laboratory Animal Unit, Nanjing Agricultural University. The mice were killed by cervical dislocation. This study was specifically approved by the Committee of Animal Research Institute, Nanjing Agricultural University, China.

### Animals and cell culture

Ten IU of pregnant mare serum gonadotropin (PMSG; Ningbo Second Hormone Factory, China) were injected intraperitoneally to three-week-old female ICR mice (Qing Long Shan Co., China) in order to induce superovulation. After 48 h, all mice were killed by cervical dislocation and their ovaries were collected for further in vitro experiments. Ovaries were placed in a culture dish with phosphate-buffered saline (PBS; Gibco, USA) and the follicles were punctured with a needle under a surgical dissecting microscope (Zeiss, Germany) in order to release MGCs. The MGCs were divided into T25 flasks, 6-well plates or 12-well plates containing Dulbecco Modified Eagle medium/F-12 (1:1; Invitrogen, USA) with 15% (v/v) fetal bovine serum (FBS; Sigma, USA) and incubated in 5% CO_2_ at 37°C for about 5 days.

### JNK activity assay

MGCs were cultured in T25 flasks (3x10^6^ cells) and treated with 20 μM SP600125 (TOCRIS Co, United Kingdom) for 12 h and with 100 μM H_2_O_2_ for 24 h. The activity of JNK was assayed by a cell JNK kinase activity assay kit (GENMED, China) according to the manufacturer's protocol. The absorbance was measured at 340 nm by using a Multiskan GO Microplate Spectrophotometer (Thermo Scientific, USA) and recorded 6 times at 1-min intervals.

### TUNEL assay

MGCs were cultured in six-well plates (1×10^6^ cells per well) and treated with 20 μM SP600125 (TOCRIS Co, United Kingdom) for 12 h and with 100 μM H_2_O_2_ for 24 h. MGCs apoptosis were detected using TUNEL staining. The staining process was carried out according to the protocol of the In Situ Cell Death Detection Kit (Roche, Germany). Samples were examined under a laser-scanning confocal microscope (Zeiss, Germany).

### Quantitative RT-PCR (qRT-PCR)

Total RNA was isolated using TRIzol (Invitrogen, USA) and reverse transcription was conducted using the PrimeScript RT Master Mix Kit (Takara, China). Quantitative RT-PCR was performed with AceQ qPCR SYBR Green Master Mix (Vazyme, China) in a reaction volume of 20 μl. The cycling parameter is as follows: 95°C for 5 min followed by 40 amplification cycles each comprising of 95°C for 10 s; 60°C for 30 s. Primer sequences are listed in S1 Table.

### Western blot

MGCs were lysed in RIPA lysis buffer (Sigma, USA) and protein concentrations were determined by BCA Protein Assay Kit (Beyotime Biotechnology, China). Total cell lysates containing 15 μg protein were loaded onto 10% SDS-PAGE gel (Jiancheng, China), separated by electrophoresis, and transferred to polyvinylidene difluoride membranes (Millipore, USA). The membranes were blocked with 5% bovine serum albumin (BSA; Sigma, USA) for 1.5 h at room temperature and were then incubated with the primary antibodies against FoxO1 (SantaCruz Biotechnology, USA), 14-3-3, Actb, or TBP (Cell Signaling Technology, USA) overnight at 4°C. After washing 3 times with TBST, membranes were incubated with horseradish peroxidase-conjugated secondary antibodies (Cell Signaling Technology, USA) for 1 h at room temperature. The membranes were visualized with an enhanced chemiluminescence detection system (LAS-4000 imager, Japan) and analyzed using ImageJ 1.42q software (National Institutes of Health, USA).

### Cell transfection and luciferase activity assay

FoxO1 expression vectors (pcDNA3-FLAG-FKHR) were kindly provided by Dr. Haojie Huang (University of Minnesota, Minnesota, USA). A 2048-bp fragment of the FoxO1 promoter containing a FRE (AGTAAACAAA) and mutations FRE site (AGTTCCGAAA) were cloned into pGL3-Basic plasmid and named pGL3-FoxO1 (W) and pGL3-FoxO1 (M) respectively, as previously described [1]. MGCs were transfected with the vectors using Lipofectamine 2000 reagent (Invitrogen, China). Luciferase activity was examined with a GloMax 20/20 luminometer (Promega, USA) 24 h after transfection. Renilla luciferase (pRL-TK) activity was used to normalize Firefly luciferase activity.

### Immunofluorescence

MGCs were cultured on coverslips in 12-well plates (4×10^5^ cells per well) and transfected with the FoxO1 expression vector, then treated with 20 μM SP600125 (TOCRIS Co, United Kingdom) for 12 h and with 100 μM H_2_O_2_ for 24 h. After washing with PBS, MGCs were fixed with 4% paraformaldehyde in PBS at room temperature for 1 h. The cells were permeabilized with 0.5% Triton X-100 solution for 10 min at 4°C and blocked with 1% BSA for 1 h at room temperature. MGCs were incubated with anti-FoxO1 antibody (1:100) for 1 h at 37°C and then incubated using a fluorescein-labeled secondary antibody (1:2000) for 1 h at room temperature under conditions of darkness. The slides were then subjected to a 20-minute nuclear staining with DAPI (Invitrogen, China) solution. Finally, the cells were observed using a laser-scanning confocal microscope (Carl Zeiss, Germany).

### Coimmunoprecipitation (Co-IP) assay

The coimmunoprecipitation assay was performed according to the manufacturer’s protocols in the Pierce Co-Immunoprecipitation Kit (Thermo, USA). After washing with 1X Modified Dulbecco’s PBS, MGCs were lysed in IP Lysis/Wash Buffer and the cell lysate was then added to the column containing the antibody. The columns were incubated with gentle rocking for 1–2 h and centrifuged. Elution Buffer was added to the columns, and the columns were centrifuged again. The flow-through was collected for further analysis by SDS-PAGE method.

### Statistics analysis

Statistical analysis was performed using SPSS v20.0 software (SPSS Inc., USA). Student’s *t*-test was used to analyze the differences between the two groups. All values were presented as mean ± SEM. *p* values < 0.05 were considered significant. All experiments were performed at least in triplicate.

## Results

### JNK is involved in MGCs apoptosis induced by oxidative stress

**Fig 1:**
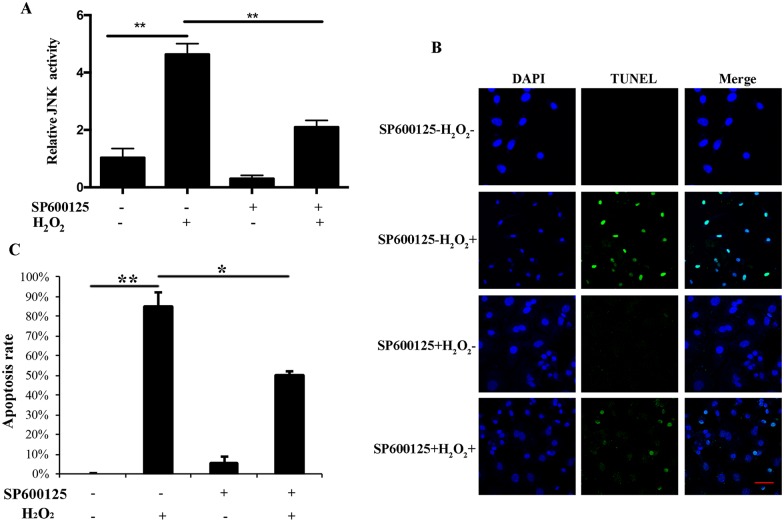
JNK mediated H_2_O_2_-induced apoptosis in MGCs. (A) JNK activation is detected after treatment with 20 μM SP600125 for 12 h and 100 μM H_2_O_2_ for 24 h. (B) MGCs are treated with 20 μM SP600125 for 12 h and then exposed to 100 μM H_2_O_2_ for 24 h. Cell apoptosis is analyzed by TUNEL assay. Apoptotic nuclei and total nuclei show green and blue fluorescence, respectively. Bar, 20 μm. (C) Quantification of the results shown in Fig 1B. All experiments are performed in triplicate. All results above represent mean ± SEM (n = 3). * *p* < 0.05; * * *p* < 0.01.

In order to confirm whether JNK is involved in oxidative stress-induced apoptosis of granulosa cells, the JNK activity after treating MGCs with H_2_O_2_ was detected. The result indicated that the JNK activity was significantly enhanced after MGCs treated with 100 μM H_2_O_2_; in contrast, JNK activity was significantly decreased by JNK inhibitor in oxidative stress-induced MGCs (Fig 1A). Moreover, we examined the MGCs apoptosis induced by H_2_O_2_ when JNK was inhibited. We found that H_2_O_2_ significantly increased the apoptosis of MGCs and the inhibition of JNK significantly decreased the apoptosis of MGCs induced by H_2_O_2_ (Fig 1B and 1C).

### JNK promotes FoxO1 translocation to the nucleus in MGCs induced by oxidative stress

**Fig 2:**
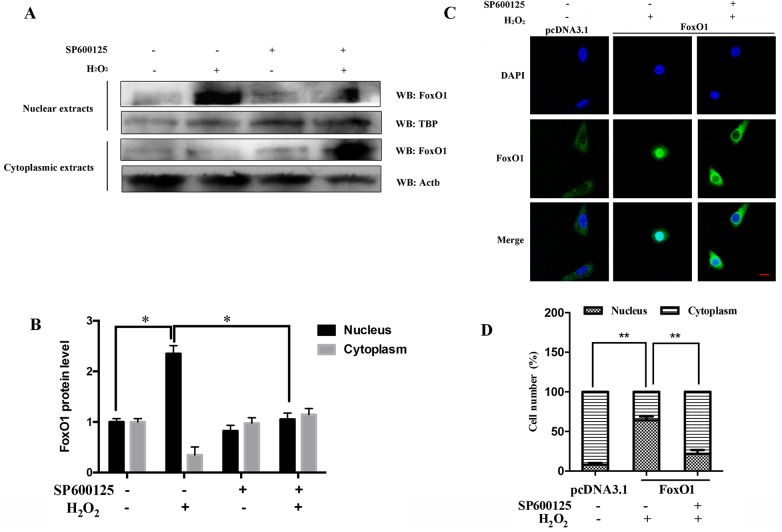
Oxidative stress promotes FoxO1 nuclear localization by mediation of the JNK activity. (A) MGCs are treated with 20 μM SP600125 for 12 h and then exposed to 100 μM H_2_O_2_ for 24 h. The expression of FoxO1 protein in cell cytoplasm and nucleus is examined using western blot. Internal controls are TBP in the nucleus and Actb in the cytoplasm. (B) Quantification of relative FoxO1 protein level. The level of expression of Actb or TBP is used to normalize the relative expression level of FoxO1 protein. (C) MGCs transfected with pcDNA 3.1 or FoxO1 expression vectors are treated with 20 μM SP600125 for 12 h and then exposed to 100 μM H_2_O_2_ for 24 h. Immunofluorescence subcellular localization of FoxO1 is identified using anti-FoxO1 (green), and the nuclei are counterstained with DAPI (blue). Bar, 20 μm. (D) The statistical analysis of FoxO1 in the nucleus and in the cytoplasm. All results above represent mean ± SEM (n = 3). * *p* < 0.05; * * *p* < 0.01.

To investigate the hypothesis that the activation of JNK affects the location of FoxO1 in the MGCs, we first examined the expression of FoxO1 protein in the nucleus and cytoplasm by western blot. The nuclear and cytoplasmic FoxO1 proteins were isolated from the MGCs after treatment with H_2_O_2_. The result showed that the expression of nuclear FoxO1 protein was higher after treatment with H_2_O_2_ (Fig 2A and 2B). However, the expression of nuclear FoxO1 protein obviously decreased when JNK was inhibited in MGCs (Fig 2A and 2B). Moreover, we examined the distribution of FoxO1 in the nucleus and cytoplasm by immunofluorescence. We first overexpressed the FoxO1 in MGCs and then used SP600125 and H_2_O_2_ to treat MGCs. The result indicated that the overexpression vector of FoxO1 significantly increased the expression of FoxO1 compared with pcDNA3.1 (Fig 2C). In addition, the immunofluorescence assay showed that FoxO1 was localized mostly in the cytoplasm after the treatment with SP600125 (Fig 2C and 2D).

### JNK promotes the dissociation of FoxO1 from 14-3-3 proteins under oxidative stress

**Fig 3:**
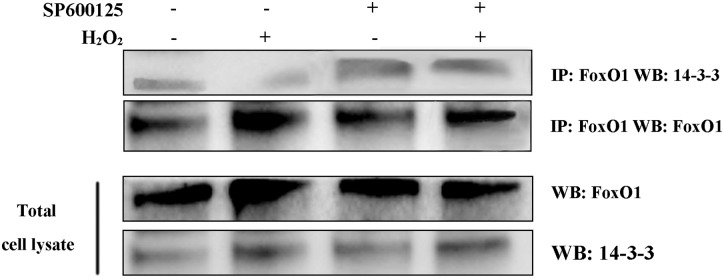
JNK affects the interaction between FoxO1 and 14-3-3 proteins in response to oxidative stress. MGCs are treated with 20 μM SP600125 for 12 h and then exposed to 100 μM H_2_O_2_ for 24 h, following precipitation with FoxO1 antibody. Precipitates are loaded for SDS/PAGE analysis and transferred for western blotting with FoxO1 and 14-3-3 proteins antibody.

As shown in a previous study, the activation of JNK can participate in the phosphorylation of Ser186 site 14-3-3 proteins, thereby interfering with the cytoplasmic localization of FoxO1 in other cells [28]. To confirm whether JNK affects the interaction between FoxO1 and 14-3-3 proteins, we used the Co-IP to detect this interaction in the MGCs. Results showed that oxidative stress induced the dissociation between FoxO1 and 14-3-3 proteins (Fig 3). In contrast, when the JNK activity was inhibited by SP600125, oxidative stress could not induce the dissociation between FoxO1 and 14-3-3 proteins (Fig 3).

### Oxidative stress regulates the expression of FoxO1 via JNK/FoxO1 axis and its self-regulation

**Fig 4:**
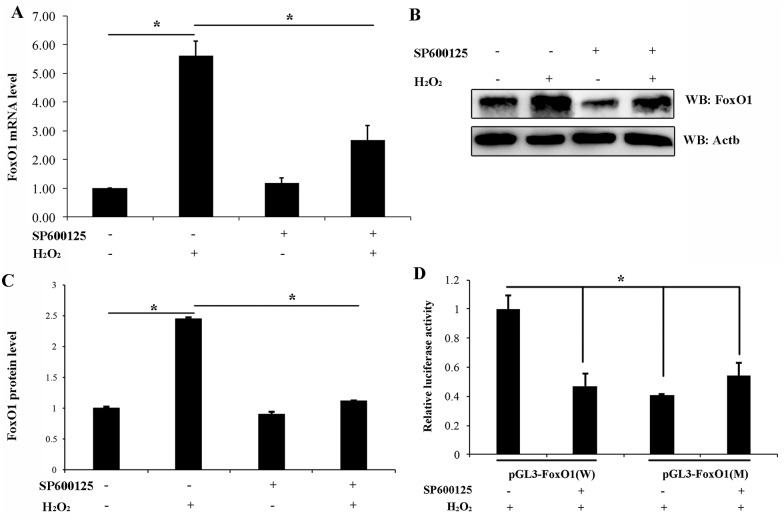
Oxidative stress regulates FoxO1 expression through its self-regulation. (A) MGCs are treated with 20 μM SP600125 for 12 h and then exposed to 100 μM H_2_O_2_ for 24 h. The mRNA expression of FoxO1 is detected in MGCs and normalized by the expression level of Actb. (B) FoxO1 protein level in MGCs after treatment with 20 μM SP600125 for 12 h and 100 μM H_2_O_2_ for 24 h. Actb serves as the internal control. (C) Quantification of relative FoxO1 protein level. The relative protein level of FoxO1 is normalized by the expression level of Actb. (D) MGCs are transfected with pGL3-FoxO1 (W) or pGL3-FoxO1 (M) for 36 h, treated with SP600125 for 12 h, and exposed to 100 μM H_2_O_2_ for 24 h. Activities are normalized using those of pRL-TK. All results above represent mean ± SEM (n = 3). * *p* < 0.05.

A previous study showed that the expression of FoxO1 increased in the MGCs treated with H_2_O_2_ [29], and so, we suspected that oxidative stress induced the FoxO1 expression via JNK pathway in MGCs. Therefore, the FoxO1 expression was analyzed in H_2_O_2_-treated MGCs when the JNK activity was inhibited by SP600125. FoxO1 mRNA and protein levels in the H_2_O_2_-treated group significantly increased compared with the control group (Fig 4A–4C), which was consistent with the results of the aforementioned study. Furthermore, when the JNK activity was inhibited by SP600125, FoxO1 mRNA and protein levels were significantly lower than those in the control group (Fig 4A–4C).

In the present study, we found that JNK was involved not only in the process of FoxO1 migrating to the nucleus but also in the expression of FoxO1. A previous study suggested that FoxO1 enhanced its expression by binding to FRE site within its promoter [30]. To investigate whether JNK could regulate the FoxO1 self-regulation, we transfected the FoxO1 promoter with a luciferase reporter construct containing FRE site and used the SP600125 and H_2_O_2_ to treat MGCs. Results indicated that FoxO1 significantly enhanced its promoter activity in the FoxO1 wild-type (W) vector containing FRE site compared with the FoxO1 mutation-type (M) vector containing a mutation of FRE site in response to oxidative stress (Fig 4D). Notably, we observed a significant decrease in luciferase activity in MGCs of the JNK inhibitor group compared with the control group (Fig 4D).

## Discussion

Previous studies showed that detrimental effects of oxidative stress can affect female reproduction and development [31, 32]. In this study, we found that oxidative stress can induce MGCs apoptosis in vitro. The mitogen-activated protein kinase (MAPK) superfamily contains three members of classical MAPK (also named ERK), JNK and p38 [33]. ERK and p38α MAPK play an important role in oocyte meiotic maturation [33, 34] and oocyte activation [35]. A previous study showed that JNK is involved in the phosphorylation of T451 and T447 in FoxO4 after the treatment with H_2_O_2_ [36]. In RAW cells, cypermethrin induced cell apoptosis via the activation of JNK and ERK MAPK signaling pathways [37]. 2-Methoxy-6-acetyl-7-methyl juglone (MAM) also induced cell death mediated in part by JNK activation in human umbilical vein endothelial cells (HUVECs) [38]. Cao et al. [39] reported that sesamin inhibited the fluoride-induced apoptosis by reducing the level of p-JNK in carp kidney. Moreover, isoliensinine-induced apoptosis in human breast cancer cells was mediated by p38 MAPK and JNK pathways [40]. The level of p-JNK increased in methotrexate (MTX)-treated platelet apoptosis, and it activated Bad and Bax and inhibited Bcl-2, thus, contributing toward mitochondrial dysfunction [41]. Serum-free media could increase the H_2_O_2_ production and activate JNK in MLO-Y4 osteocyte-like cells, resulting in cell apoptosis [42]. In the present study, the JNK activity significantly increased in H_2_O_2_-treated MGCs. Results showed that JNK is involved in the regulation of the oxidative stress-induced cell apoptosis in MGCs. Furthermore, SP600125 inhibited the H_2_O_2_-induced MGCs apoptosis. Taken together, these results suggested that JNK plays an important role in the process of H_2_O_2_-induced apoptosis in MGCs.

Oxidative stress promotes FoxO nuclear translocation and induces cell apoptosis through the activation of FoxO transcription factors in neurons [43]. In addition, FoxO transcription factors induced cell apoptosis by targeting the pro-apoptotic genes, including *Puma*, *Bim*, *TRAIL*, and *FasL* [20, 44]. We observed that oxidative stress enhanced the expression of FoxO1 in the nucleus and promoted its translocation to the nucleus in MGCs. These results suggested that oxidative stress induces FoxO1 nuclear import, resulting in MGCs apoptosis. JNK-dependent threonine 447 and threonine 451 phosphorylation promoted the nuclear translocation and increased the transcriptional activity of FoxO4 [36]. An excessive accumulation of cellular ROS induced the expression of *Bim* and *Puma* via JNK/FoxO signaling in mouse embryonic stem (mES) cells [45]. In *Caenorhabditis elegans*, JNK-1 promoted the nuclear accumulation of DAF-16 in response to heat stress [28]. In the present study, the nuclear translocation of FoxO1 induced by oxidative-stress was inhibited by SP600125, suggesting that JNK mediates oxidative stress-induced cell apoptosis via the nuclear translocation of FoxO1 in MGCs.

MST1 phosphorylated FoxO3 at serine 207 and disrupted the association of FoxO3 with 14-3-3 proteins, promoting the translocation of FoxO3 to the nucleus [43]. Moreover, Yuan et al. [46] found that MST1-induced phosphorylation of FoxO1 at serine 212 dissociated FoxO1 from 14-3-3 proteins and mediated the nuclear translocation of FoxO1 in primary rat cerebellar granule neurons. The vasoactive peptide urotensin-II (U-II) decreased the interaction of FoxO3 with 14-3-3 proteins in stimulated smooth muscle cells (SMCs) of human [47]. We also observed the dissociation of FoxO1 from 14-3-3 proteins in MGCs under oxidative stress. This showed that oxidative stress increases the nuclear translocation of FoxO1 through the disruption of FoxO1 from 14-3-3 proteins, leading to cell apoptosis. The JNK-mediated phosphorylation of 14-3-3 proteins contributed to the disruption of FoxO3a from 14-3-3 proteins [28]. A previous study in SMCs showed that U-II activated the phosphorylation of 14-3-3 proteins, while the JNK inhibitor SP600125 attenuated it [47]. In the present study, SP600125 increased the interaction between FoxO1 and 14-3-3 proteins in oxidative stress-induced MGCs, suggesting that JNK is required for the oxidative stress-mediated dissociation of FoxO1 with 14-3-3 proteins. Taken together, oxidative stress reduces the association of FoxO1 with 14-3-3 proteins and the inhibition of JNK restores this association.

A recent study showed that hypoxia significantly increased the expression of FoxO3a in CMECs [48]. In the present study, oxidative stress also enhanced the expression of FoxO1 in MGCs, which is consistent with the results of the study by Shen et al. [1]. Notably, when JNK was inhibited in MGCs, FoxO1 expression decreased in response to the oxidative stress. This result showed that JNK affected the expression and nuclear translocation of FoxO1 in oxidative stress-induced MGCs. A previous study showed that FoxO1 bound directly to the FoxO1 promoter, inducing its own expression [30]. Moreover, FoxO3 regulated the expression of FoxO1 by binding to its promoter in HEK293T cells [49]. We observed that oxidative stress stimulated the transcriptional activity of FoxO1 in MGCs. In contrast, SP600125 decreased the transcriptional activity of FoxO1 under oxidative stress. These results indicated that JNK promotes the nuclear translocation of FoxO1 inducing its transcriptional activity via self-regulation in oxidative stress-induced MGCs.

## Supporting Information

S1 Table Primers used in this study. (DOC)
