# Supplementary material for: Oxidative Stress Induces Mouse Follicular Granulosa Cells Apoptosis via JNK/FoxO1 Pathway
Source: PLoS One. 2016 Dec 9;11(12):e0167869. doi: 10.1371/journal.pone.0167869 (PMC5148000; doi:10.1371/journal.pone.0167869)
Supplement: S1 Table — (DOC) [file pone.0167869.s001.doc]

**S1 Table Primers used in this study**

| Gene name | GenBank  No. | Primer squence（5’-3’） | Annealing temp (°C) | Product size （bp） |
| --- | --- | --- | --- | --- |
| *FoxO1* | [NM_019739.3](https://www.ncbi.nlm.nih.gov/entrez/viewer.fcgi?db=nucleotide&id=239985491) | F: CGTGCTTACAGCCTTCTA  R: ACCTCCATCGTGACAAAA | 60 | 181 |
| *Actb* | [NM_007393.5](https://www.ncbi.nlm.nih.gov/entrez/viewer.fcgi?db=nucleotide&id=930945786) | F: GCTGTCCCTGTATGCCTCT  R: GTCTTTACGGATGTCAACG | 60 | 460 |
